# No Intern Left Behind: Using a Dedicated Transition to Discipline Block Improves Resident Outcomes

**DOI:** 10.15694/mep.2020.000065.1

**Published:** 2020-04-03

**Authors:** Robert Battisti, Briana Donaldson, Perry Lin

**Affiliations:** 1 Mount Carmel Grove City

**Keywords:** Curriculum, Evaluation, Internal medicine, Remediation, Critical Deficits, ACGME, Milestones Ratings

## Abstract

This article was migrated. The article was marked as recommended.

**Introduction:** A program was developed for incoming PGY-1 residents using the Accreditation Council for Graduate Medical Education milestones ratings. This program detects critical deficiencies and works to correct them as early as possible.

**Methods:** A one month period was used for this transition to discipline block to identify at-risk learners. The block utilized cased-based discussions, interactive lectures, simulations, and clinical core rotations. All activities were tied to milestones measures to recognize deficiencies and provided a goal to correct the individual’s progression.

**Results:** Interns that completed the transition to discipline block were compared to the most recent previous class at the same institution. The same number of individuals with critical deficits were found in each class at first milestones rating (4 deficits per class, p value 1.0). The intervention classes had critical deficiencies recognized earlier and all identified deficiencies were extinguished earlier. Medical knowledge as compared by In-Training Examination percentile scores improved (Pre-Intervention Mean Percentile 28.9, Post-Intervention Mean Percentile 49.5, p value 0.005).

**Discussion:** A milestones-based transition to residency block identified critical deficiencies earlier, which allowed for earlier intervention and improvement in resident performance. A similar process may benefit other residency programs.

## Introduction

The American medical education system has transitioned towards a competency-based education as the foundation for undergraduate and graduate medical education ( Accreditation Council for Graduate Medical Education, 2018; American Board of Medical Specialties, 2019). Despite a more unified framework for expectations and assessment, the transition from senior medical student to post-graduate year one (PGY-1) is complex and stressful for learners ( Willcock *et al.*, 2004). It may even be associated with higher mortality of patients ( Young *et al.*, 2011). As the breadth of medical knowledge medical schools are responsible for imparting continues to expand, schools must work even more diligently to address the needs of all of their graduates. However, many graduates lack the skills and knowledge necessary to succeed in residency ( Langdale *et al.*, 2003; Lyss-Lerman *et al.*, 2009). In fact, only 53.8% of residents agree that their medical school prepared them to excel in residency ( Chen, Kotliar, and Drolet, 2015). PGY-1 residents enter residency at different levels of competency in different areas due to their disparate medical school experiences. Often times, the program directors and faculty are not fully aware of the readiness level until several months into training.

The expectations program directors’ have of PGY-1 resident’s skills are often not reflective of the actual skills of new trainees ( Raymond *et al.*, 2011). To address these issues, The Royal College of Physicians and Surgeons of Canada launched their Competence by Design program in 2017, this program includes a mandatory transition to discipline block for new medical school graduates ( Oswald and Abbot, 2017). No similar program is currently required in the U.S. Medical Education system.

Transition to residency curriculums for medical students have been shown to ease the transition into residency by helping learners understand their own shortcomings and develop self-guided learning to overcome deficits ( Brownfield, Wong, and Blue, 2016; Laack, Newman, Goyal, and Torsher, 2010; Teo *et al.*, 2011. These courses are becoming more common in medical schools, but are still not present as often among residency training programs. Immersion blocks to address gaps in perceived versus actual skills have been utilized by residency training programs. A meta-analysis ( Blackmore *et al.*, 2015) showed that such graduate medical education courses demonstrated large improvements in clinical skills and confidence. Other previous work has shown earlier competency in patient management and procedural skill within surgical residency with the inclusion of a preparatory course ( Wunder, Brandt, and Lipman, 2018).

To assess the developmental outcomes of residents the Accreditation Council for Graduate Medical Education (ACGME) requires that programs evaluate their trainees semi-annually using a specialty specific competency-based rating system to track the resident’s progress, referred to as milestones ratings ( Holmboe, Edgar, and Hamstra, 2016). Generally, Internal Medicine residents progress from lower to higher milestone ratings over the course of residency, but do not progress uniformly, or linearly over time ( Warm *et al.* 2016). These ratings are given semi-annually by the program’s Clinical Competency Committee (CCC). Under current requirements, the first mandatory milestones rating report occurs after nearly 6 months of training ( Holmboe, Edgar, and Hamstra, 2016). This lag can result in delayed identification of critical deficiencies among residents. Identification of critical deficiencies in ACGME Milestones ratings is not rare among medical trainees, and this identification is vital to the development of remediation plans ( Kinnear *et al.*, 2017). Any delay in identification hinders effective implementation of a remediation process for struggling learners as, in our experience, bad habits often become ingrained behaviors after several months.

It has been demonstrated ( Sozener *et al.*, 2016) that a post-match milestones based assessment of medical students was valuable to Emergency Medicine program directors, as an “educational handover.” The value of early identification of deficits was of great interest to program directors. We are not aware of a publication wherein the residency program itself did this early assessment, coupled with training (including local culture and needs) of new trainees.

## Methods

Mount Carmel’s Internal Medicine Faculty developed an immersive, intensive, one month long transition to discipline block as the initial rotation for our new PGY-1 residents. We began utilizing this block in July of 2017 and have continued annually through July 2019, as the first rotation for our new PGY-1 residents.

This block is designed to reinforce knowledge with an emphasis on urgent and emergent situations, while also specifically addressing important psychosocial, cultural and professional issues. Skills addressed include dealing with the death of patients, developing rapport with patients, and time management. These skills reflect topics residents have reported feeling less prepared for after medical school ( Young *et al.*, 2011).

During this time we also build in several wellness half-days, during which the new residents are given recommendations for and encouraged to seek primary care, dental care, or encouraged to complete their move-in process with utilities, driver’s license change, etc. During this block, our new residents also have the opportunity to begin to participate in guided group reflection sessions with a professional psychologist. Individual counseling sessions are also available with this psychologist to promote wellness longitudinally throughout training. For sample schedules, please see Supplementary Files A1-A5.

The curriculum undergoes iterative improvement each year based upon deficits identified in incoming residents from previous years as well as feedback to the effectiveness of the curriculum. Residents and faculty are both given the opportunity to provide narrative feedback. This feedback has led to the development of new educational curriculum, development of more engaging classroom activities using clinical cases, and changes in how simulation is created and delivered.

To promote retention of topics related to urgent and emergent care, those topics were covered multiple times in various modalities over the course of a week, as spaced repetition has been shown to enhance assimilation of material ( Kang, 2016). Typically, reading material (either from common internal medicine board preparatory courses, sentinel articles, or review articles) was provided to the learners. This material was then discussed in a case-based question and answer format. We utilized a team approach to problem-solving, differential diagnosis building, and treatment decisions to help enhance team building. At the end of the week, these topics were tested in a simulated environment in an individual or group setting. The simulation environment allows for observed assessment by the faculty of the learners’ understanding and ability to apply the information related to an urgent or emergent scenario.

Simulated environments have demonstrated improvement in medical knowledge, increased comfort in procedures, and enhanced performances during retesting in simulated scenarios. Simulation has also been shown to be a reliable tool for assessing learners for teamwork and communication skills ( Okuda *et al.*, 2009). Performing this assessment in a simulated patient environment allows for direct manipulation of single variables to assess sub-competencies of internal medicine that are not often directly observed during early stages of residency. These sub-competencies include: working effectively with multidisciplinary team (SBP1), learning and improving via feedback (PBLI3), learning and improving at the point of care (PBLI4), and communicating effectively in inter-professional teams (ICS2). The simulated environment provided another unique feature by allowing new residents to give each other feedback while permitting us to see the learner’s response to feedback and future incorporation.

Simulations allow us to observe the reaction of our learners, providing faculty the opportunity to engage in mentoring and advice for future real world situations. Each session, classroom-based and simulation-based, was tied to 6-10 of the Internal Medicine milestone sub-competencies. Over the course of the block, 477 total sub-competency ratings on each individual learner were compiled. For each activity, the faculty member leading the session was asked to record in a spread sheet whether a critical deficiency was or was not present for each PGY-1 resident for each associated sub-competency linked to the activity. A critical deficiency is defined by the ACGME as a rating of 1 on a sub-competency ( Oswald and Abbot, 2017). Written feedback was compiled for below expectations or exceeding expectations ratings during these activities. For a sample feedback form please see Supplementary File B.

Learners in whom a critical deficiency was identified were given formative feedback for each deficiency. Every deficiency was tied to a specific activity, allowing the feedback to include the precise action which caused a below expectations rating for their level of training. For any critical deficiency discovered, an individualized action plan was developed to address the deficiency. Faculty were informed of all deficiencies and subsequent action plans. This process occurred during the introductory block resulting in development of remediation plans prior to residents beginning their first full clinical rotation.

Six months after intern school we evaluate the curriculum again. This evaluation timing coincides with the CCC requirement for the first competency evaluation of residents. This evaluation involves a team-based review of milestones achieved by PGY-1 learners as rated by the CCC which is based on a review of the summative narrative evaluation as well as review of quantitative data. The goal of this session is to evaluate the effectiveness of newly introduced topics and changes made to the curriculum. Changes made to the curriculum are based on the reflection of areas of positive improvement and areas where opportunities to improve still exist. An effort is made to eliminate material that has shown to have negligible improvement or, in rare cases, regression. After this six month evaluation, planning for the upcoming year and changes to the curriculum begin.

We tracked the findings of critical deficiencies among the intervention classes against matched classes prior to the intervention. The intervention classes had their initial milestones rating at one month, while the control class had their initial milestones rating at 6 months, in line with usual ACGME requirements. We assessed the control class at six month intervals per usual ACGME requirements. The intervention classes were assessed at the usual ACGME required six month intervals in addition to the initial assessment during the first month. The differences were analyzed by Chi-Squaredanalysis. The control class had all ratings done by the CCC, the membership of which remained consistent for the ratings of the intervention classes as well. Ratings performed prior to this class were performed by different faculty members and discarded due to concern for inter-rater reliability.

In an effort to assess whether this program objectively impacted medical knowledge, we also tracked the PGY-1 Internal Medicine In-Training Exam (ITE), administered by the American College of Physicians. This examination is administered in August and September nationally to all Internal Medicine residents. It provides and unbiased comparison of their medical knowledge to their peers nationally. The PGY-1 ITE scores were then analyzed by student’s T-test for comparison. The first two matched classes before intervention were compared to the two intervention classes for this comparison.

## Results

At initial assessment of the control class (time six months), we found that four of the eight (50%) PGY-1 residents had critical deficiencies found. Some residents were found to have multiple critical deficiencies. Overall, there was at least one critical deficiency in each of the five competency areas. No specific pattern of deficiencies was noted.

At initial assessment for the intervention classes (time one month) we found that eight of 16 (50%) PGY-1 residents in the two separate intervention classes had critical deficiencies. Again, some residents were found to have multiple deficiencies, and four of the five areas of competency were found to account for at least one critical deficiency. No pattern of critical deficiencies was noted, and there was a disparate assortment of critical deficiencies found in each class.

**Figure 1.**
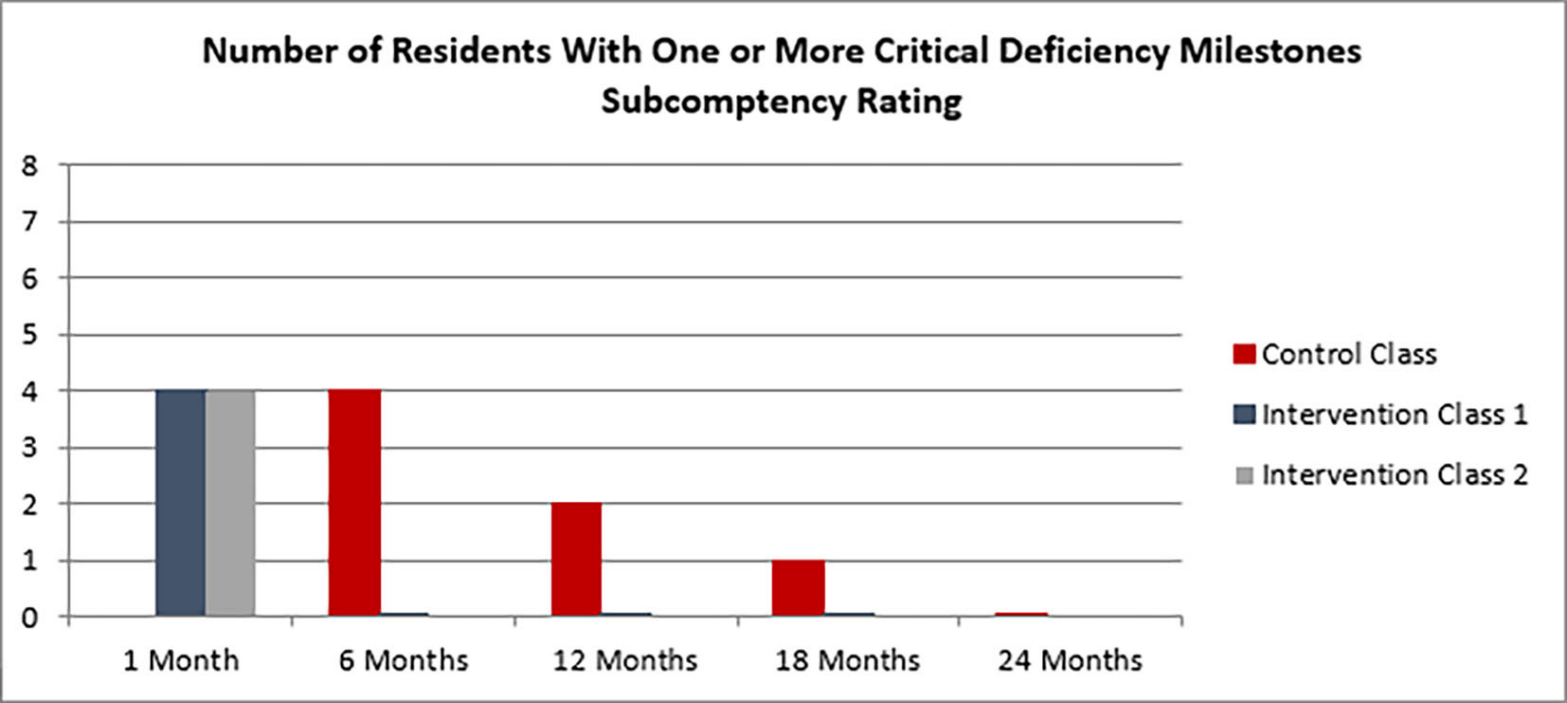
Graph of Residents with critical deficiency ratings over time in training.

Despite similar initial assessments, interns in the control class were found to have critical deficiencies persisting in future milestones ratings, including to as far out as 18 months into training, with extinguishing of critical deficits not being realized until 24 months of training. Those learners in the intervention classes, however, extinguished all critical deficiencies by their 6 month rating (see figure 1). The intervention classes have not had a recurrence of any critical deficiencies to date, however they are still in training at the time of writing of this manuscript.

At initial assessment the same number of residents from each class were found to have critical deficiencies. Those in the intervention group extinguished critical deficiencies by their 6 month rating and they did not re-emerge.

**Figure 2.**
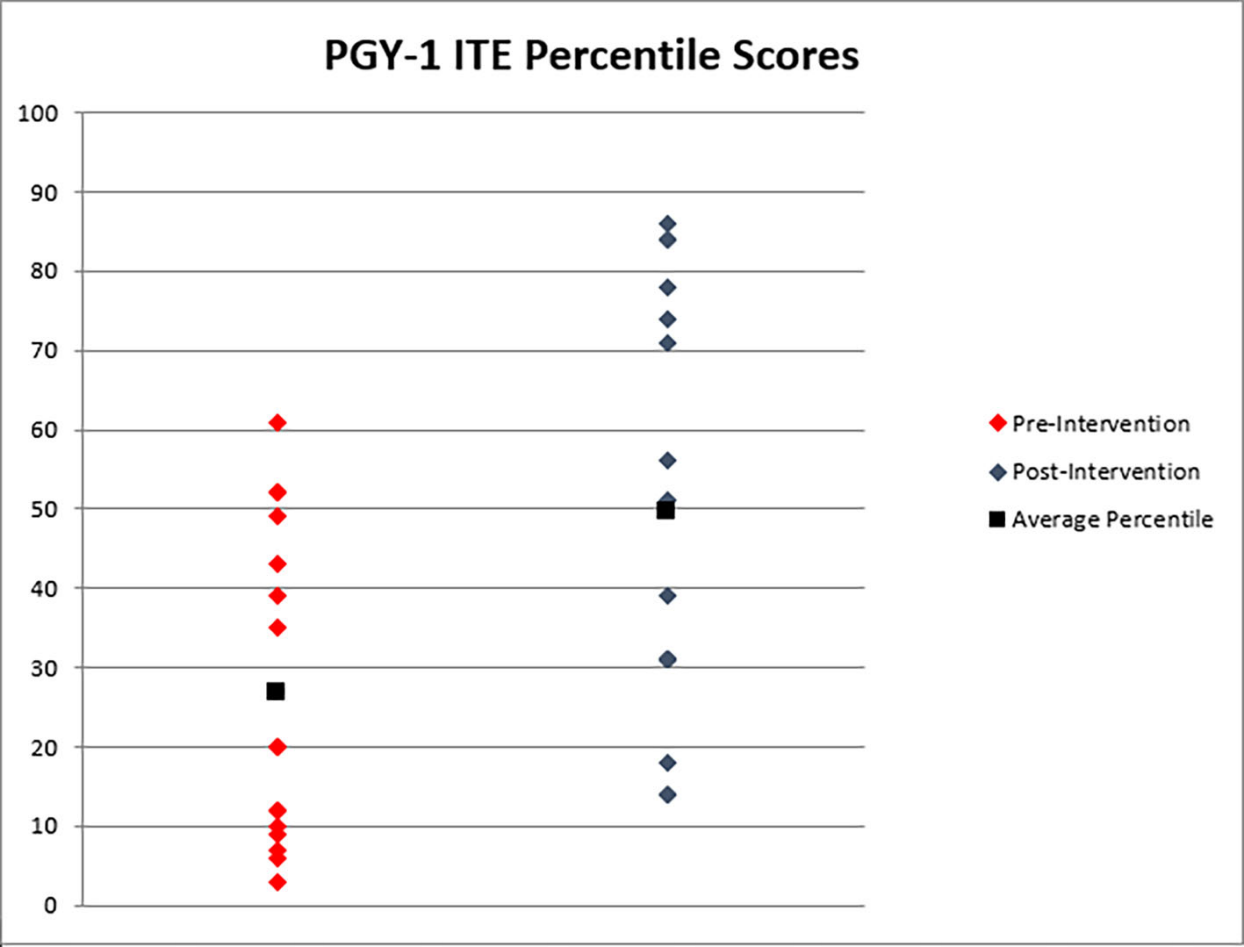
Individual and mean percentiles of the two most recent pre-intervention (control) classes compared to the two intervention classes.

The two control classes had a mean PGY-1 ITE percentile of 28.9 (range 3-61). The two intervention classes had a mean ITE percentile of 49.5 (range 14-86) (See figure 2 for all scores). The difference of those mean scores are statistically significant with a p-value of 0.005. There was no change during this interval in our programmatic board preparation or education for standardized exams.

## Discussion

Our results show that a transition to discipline introductory rotation block can identify critical deficiencies early in training. We have also shown that this early identification of critical deficiencies can lead to earlier correction. We hypothesize that this is because the behaviors leading to critical deficits have not had time to become entrenched or routine. Such critical deficits are relatively common among internal medicine trainees, and identification is critical in developing a plan for eradicating the deficits. By showing learners as early as possible in the course of their residency training that these deficits exist and by developing individualized remediation plans for them, we have found deficits can be successfully extinguished early in training.

We built upon existing literature showing medical knowledge and its application can be improved by an introductory or “bootcamp-style” block. We believe that when tied to a milestones based assessment, and provided by the residency faculty, this transition to discipline block is more supportive of residents advancing in milestones sub-competency rating. Our intervention classes showed significant improvement in ITE scores as compared to the control classes, showing that they retained the educational component of our introductory block for at least several months. This enhanced medical knowledge and application could possibly also mitigate the “July effect,” by improving patient care and potentially reducing medical errors.

As the curriculum for medical schools must continue to expand with time and advancement in medical science, it will likely become increasingly difficult for medical schools to meet all challenges that will face new physicians in residency. We believe that this introductory transition to discipline block represents an immense opportunity for residency programs to change the trajectory of milestone ratings for new residents. By identifying deficits early, prompt corrective plans can be put in place, which will facilitate residents’ progression through the milestones at a faster rate.

There are several limitations to our work. Milestones ratings were done by the same Clinical Competency Committee across this project, but milestones ratings can be seen as subjective. We attempted to limit subjectivity as possible by using milestones ratings only from the same CCC with an established protocol.

We also used the In-Training Exam results as an objective measure of medical knowledge, however the American Board of Internal Medicine (ABIM) exam passage rate may be a better measure. At the time of the writing of this manuscript, however, the classes enrolled in this program are still in training and not yet eligible to take the ABIM exam.

This project was done at a single academic medical center, within a single specialty, and within a relatively small program, which could bring into question the applicability to other specialties or academic centers and significantly limits statistical power.

As there are differing gaps among individual students entering residency, there is a broad spectrum of areas in which education must be given. We have very little literature to use to determine the curriculum. Our curriculum is reflective of the tasks and medical knowledge asked of incoming interns at our institution. We do believe this curriculum is reflective of most Internal Medicine programs and the gaps seen most often from the medical schools. We are limited in the scope of our results by the selection of our learners being from the limited population of medical schools that we recruit from most heavily.

Due to the iterative nature of the curriculum, there is a constant shift towards varying areas of competency. In our third year, there has been a relative stabilization of topics, but there continues to be year-to-year changes for improvement. There is also a trend to ongoing entropy with an ever expansion of topics. This increasing amount of education has led to concern about the efficient frontier. There may be a point in which marginal gain in education and skill come at a high cost of lack of clinical exposure. Determination of this point has been difficult as there are no available studies to help guide this evaluation. We understand that we may, even at this time, have too many topics or cover a topic too thoroughly with time being better spent on teaching other aspects.

## Conclusion

Dedicated transition to discipline blocks can be used to address gaps in perceived versus actual skills, identify and begin correcting critical deficiencies at the beginning of residency, and improve baseline medical knowledge. We believe such a block would benefit residency training programs at large. This innovation could create a solid foundation for education and feedback, which can improve an entire generation of physicians.

## Take Home Messages

• Critical deficiencies in milestones ratings are common among internal medicine trainees. Their identification is critical to any remediation of the deficiency.
• An introductory transition to discipline block can be used for PGY-1 trainees to evaluate their knowledge, skills, and attitudes and identify critical deficiencies at the beginning of residency.
• Earlier identification of critical deficiencies leads to earlier correcting of these deficits, as behaviors do not become entrenched this way.
• A similar transition to discipline block in other programs or other institutions may improve the difficult evolution from medical student to resident, with improved resident outcomes.

## Notes On Contributors

**Robert Battisti** is an Assistant Program Director and Core Faculty member for Mount Carmel Grove City’s Internal Medicine Residency Program in Ohio.

**Briana Donaldson** is an Assistant Program Director and Core Faculty member for Mount Carmel Grove City’s Internal Medicine Residency Program in Ohio.

**Perry Lin** is an Assistant Program Director and Core Faculty member for Mount Carmel Grove City’s Internal Medicine Residency Program in Ohio.
