# Supplementary material for: No Intern Left Behind: Using a Dedicated Transition to Discipline Block Improves Resident Outcomes
Source: MedEdPublish (2016). 2020 Apr 3;9:65. [Version 1] doi: 10.15694/mep.2020.000065.1 (PMC10697443; doi:10.15694/mep.2020.000065.1)
Supplement: Peer review history

# Reviewer report: Reviewer response for version 1

**DOI:** 10.21956/mep.20003.r30480

**Published:** 2020-04-28

**Authors:** Louise Baird

**Affiliations:** 1 St George Hospital

This review has been migrated. The reviewer awarded 4 stars out of 5 This was a well written manuscript describing a new educational intervention addressing identified deficiencies in your recent PGY-1 cohorts. The methodology used sound educational pedagogy, aligning the learning activities to your educational objectives. The results showed the program was not only successful at identifying areas where new graduates were underperforming, but showed the remediation plans were successful very quickly. These findings will be of interest to those involved in training who struggle to implement rapidly successful remediation. More detail of these implementation plans could be described in this paper, or even form a separate paper. I agree with the other reviewers that medical schools need greater information on what their graduates are underperforming in to improve their programs. Miller's pyramid describes competency - 'shows how', but what we really what to know is that this is what a graduate 'does' in a real world setting. This research highlights the gaps between a medical school certifying competency and a graduate doing this in practice.

Reviewer Expertise:

NA

No decision status is available

# Reviewer report: Reviewer response for version 1

**DOI:** 10.21956/mep.20003.r30479

**Published:** 2020-04-13

**Authors:** Balakrishnan Nair

**Affiliations:** 1 Centre for Medical Professional Development and University of Newcastle

This review has been migrated. The reviewer awarded 4 stars out of 5 I enjoyed reading this article. Medical Schools should have outcome based curriculum and then design down this in a spiral manner. Often, as the authors state, residents are not prepared to meet the expectations. Many schools , including mine, are trying to introduce the final year as a clinical clerkship year and embed the student into the workplace for appropriate learning. If we do not do this , the patient will not get the care they need and the program directors will have to pick up the challenge , to get the residents up to speed.It is good to see the positive outcome of this boot camp approach. I am not sure who did the work when the residents were away learning . I note the small sample size too. It will be good to replicate the study and look at the long term outcome of this intervention as the authors state

Reviewer Expertise:

NA

No decision status is available

# Reviewer report: Reviewer response for version 1

**DOI:** 10.21956/mep.20003.r30478

**Published:** 2020-04-07

**Authors:** Yingzi Huang

**Affiliations:** 1 The First Affiliated Hospital, Sun Yat-sen University

This review has been migrated. The reviewer awarded 3 stars out of 5 I enjoyed reading this article and it’s a good example of timely remediation plan for critical deficiencies before it causes medical errors during residency. However, as medical education is a continuum, I then wonder when is the best timing for picking up the deficiencies and fix it even earlier before it’s late. If only 53.8% of residents feel they are well prepared for residency, is there anything wrong and fixable in the undergraduate level, when problems can be addressed in a relatively cost-effective way without stressing both the tutors and students/residents later on in residency, when it’s perhaps more difficult to balance work and study. Is it possible for clinical exposure and spaced repetition to start earlier, so that when they graduate, medical students are more prepared and longer term learning effect can be achieved?

Reviewer Expertise:

NA

No decision status is available
